# Aqua­(hexa­methyl­enetetra­mine-κ*N*)bis­(methanol-κ*O*)bis­(thio­cyanato-κ*N*)cobalt(II)

**DOI:** 10.1107/S160053680802357X

**Published:** 2008-08-20

**Authors:** Wei-Li Shang, Yan Bai, Chao-Zhong Ma, Zhi-Min Li

**Affiliations:** a Institute of Molecular and Crystal Engineering, School of Chemistry and Chemical Engineering, Henan University, Kaifeng, Henan 475004, People’s Republic of China

## Abstract

In the title complex, [Co(NCS)_2_(C_6_H_12_N_4_)(CH_4_O)_2_(H_2_O)], the six-coordinated Co atom has a slightly distorted octa­hedral geometry. The molecules are linked by intermolecular O—H⋯S and O—H⋯N hydrogen bonds, forming a three- dimensional crystal structure. Intramolecular C—H⋯N and C—H⋯O hydrogen bonds are also present.

## Related literature

For information on the self-assembly of transition-metal complexes, see: Guo *et al.* (2002 ▶); Kumar *et al.* (2007 ▶); Venkateswaran *et al.* (2007 ▶); Chi *et al.* (2008 ▶). For complexes including hexa­methyl­enetetra­mine (hmt) as ligand, see: Liu *et al.* (2006 ▶); Zhang *et al.* (1999 ▶); Meng *et al.* (2001 ▶); Li *et al.* (2002 ▶, 2007 ▶); Banerjee *et al.* (2007 ▶).
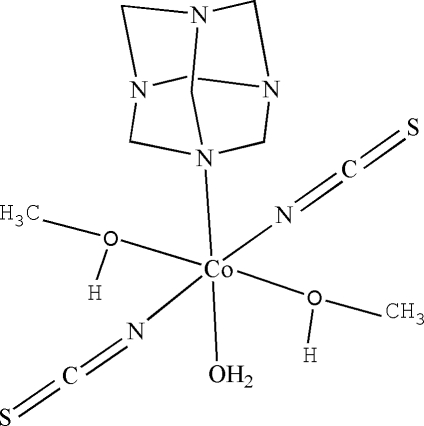


## Experimental

### 

#### Crystal data

[Co(NCS)_2_(C_6_H_12_N_4_)(CH_4_O)_2_(H_2_O)]
*M* *_r_* = 397.39
Orthorhombic, 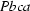

*a* = 14.1128 (8) Å
*b* = 15.3684 (9) Å
*c* = 15.9839 (9) Å
*V* = 3466.8 (3) Å^3^
*Z* = 8
Mo *K*α radiation
μ = 1.25 mm^−1^
*T* = 296 (2) K
0.30 × 0.30 × 0.25 mm

#### Data collection

Bruker APEXII diffractometer
Absorption correction: multi-scan (*SADABS*; Bruker, 2000 ▶) *T*_min_ = 0.691, *T*_max_ = 0.730
20785 measured reflections
4287 independent reflections
3528 reflections with *I* > 2σ(*I*)
*R*_int_ = 0.022

#### Refinement

*R*[*F* ^2^ > 2σ(*F* ^2^)] = 0.029
*wR*(*F* ^2^) = 0.075
*S* = 1.04
4287 reflections
217 parameters
H atoms treated by a mixture of independent and constrained refinement
Δρ_max_ = 0.64 e Å^−3^
Δρ_min_ = −0.61 e Å^−3^

### 

Data collection: *SMART* (Bruker, 2000 ▶); cell refinement: *SAINT* (Bruker, 2000 ▶); data reduction: *SAINT*; program(s) used to solve structure: *SHELXTL* (Sheldrick, 2008 ▶); program(s) used to refine structure: *SHELXTL*; molecular graphics: *SHELXTL*; software used to prepare material for publication: *SHELXTL*.

## Supplementary Material

Crystal structure: contains datablocks I, global. DOI: 10.1107/S160053680802357X/bh2177sup1.cif

Structure factors: contains datablocks I. DOI: 10.1107/S160053680802357X/bh2177Isup2.hkl

Additional supplementary materials:  crystallographic information; 3D view; checkCIF report

## Figures and Tables

**Table:** 

Co1—N2	2.0400 (15)
Co1—N1	2.0585 (15)
Co1—O2	2.1024 (13)
Co1—O1*W*	2.1268 (14)
Co1—O1	2.1760 (13)
Co1—N3	2.2785 (13)

**Table:** 

N2—Co1—N1	176.69 (6)
N2—Co1—O2	90.94 (6)
N1—Co1—O2	90.31 (6)
N2—Co1—O1*W*	89.59 (6)
N1—Co1—O1*W*	87.34 (6)
O2—Co1—O1*W*	90.09 (6)
N2—Co1—O1	89.30 (6)
N1—Co1—O1	89.35 (6)
O2—Co1—O1	178.06 (6)
O1*W*—Co1—O1	87.99 (6)
N2—Co1—N3	92.06 (6)
N1—Co1—N3	90.98 (5)
O2—Co1—N3	91.53 (5)
O1*W*—Co1—N3	177.67 (6)
O1—Co1—N3	90.39 (5)

**Table 2:** Hydrogen-bond geometry (Å, °)

*D*—H⋯*A*	*D*—H	H⋯*A*	*D*⋯*A*	*D*—H⋯*A*
C4—H4*A*⋯N1	0.97	2.52	3.119 (2)	120
C4—H4*B*⋯O1	0.97	2.56	3.167 (2)	121
C9—H9*D*⋯N2	0.96	2.56	3.181 (3)	123
O1—H1⋯N5^i^	0.80 (2)	2.08 (3)	2.824 (2)	156 (2)
O1*W*—H1*WA*⋯N6^ii^	0.76 (2)	2.07 (2)	2.821 (2)	168 (3)
O2—H2⋯N4^iii^	0.78 (2)	1.97 (2)	2.7417 (18)	172 (2)
O1*W*—H1*WB*⋯S2^iii^	0.88 (3)	2.55 (3)	3.4146 (16)	168 (2)

Symmetry codes: (i) 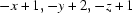; (ii) 
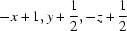
; (iii) 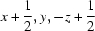.

**Fig. 1.**
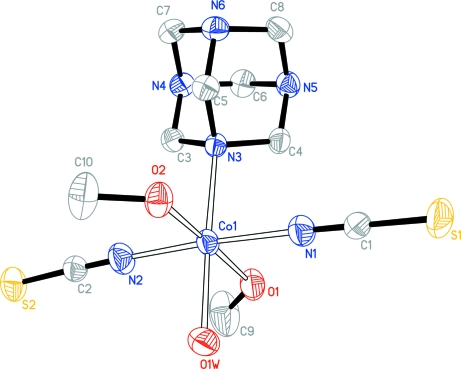
The molecular structure of the title complex, with displacement ellipsoids drawn at the 50% probability level. The hydrogen atoms are omitted for clarity.

**Fig. 2.**
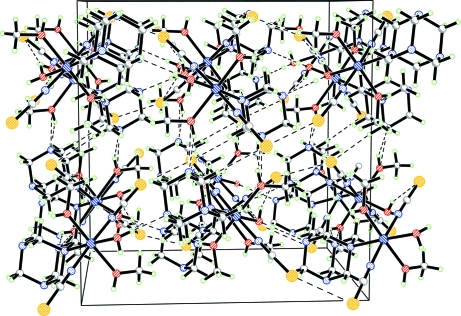
Perspective view of the three-dimensional network, showing the intermolecular hydrogen bonds (dashed solid lines) interactions.

## supplementary crystallographic information

### Comment

Much interest at present is focused on the deliberate construction of transition metal ions and organic molecules by self-assembly of the component metal complexes. These solid materials are attractive to chemists not only for the variety of topologies and intriguing frameworks, but also for their interesting properties either by strong metal-ligand bonding or by weaker bonding forces such as hydrogen bonding and π—π interactions (Guo *et al.*, 2002; Kumar *et al.*, 2007; Venkateswaran *et al.*, 2007; Chi *et al.*, 2008). Among the ligands, hexamethylenetetramine (hmt), as a potential tetradentate ligand or hydrogen bonds acceptor, seems quite suitable in self-assembly systems. Several groups have reported that Co(II), Cd(II), Mn(II) or Ni(II) complexes with hmt and SCN ^-^ as ligands form two-dimensional or three-dimensional networks (Liu *et al.*, 2006; Zhang *et al.*, 1999; Meng *et al.*, 2001; Li *et al.*, 2002; Banerjee *et al.*, 2007; Li *et al.*, 2007).

Herein, we present a new hmt complex, (I), based on Co^II^, with SCN ^-^ as ligand (Fig. 1). The title complex, which contains one cobalt center, one hmt, two NCS^-^, two coordinated methanol molecules and one coordinated water molecule, forms a mononuclear complex. The Co^II^ ion is surrounded by three N atoms and three O atoms (two N atoms from two isothiocyanates, one N atom from hmt, one O atom from coordinated water molecule and two O atoms from two methanol molecules) to attain a distorted octahedral coordination geometry. Moreover, the O atoms of both methanol molecules are each mutually *trans* to each other. Intramolecular C—H···N and C—H···O hydrogen bonds (Table 2) are important factors in the stabilization of the molecule.

In the crystal structure, molecules interact with each other, forming a three-dimensional supramolecular network through multiform intermolecular hydrogen bonds (Fig. 2 and Table 2). The O2 and O1w atoms form two O—H···N hydrogen bonds with N4 and N6 atoms of the adjacent hmt ligand, respectively. In addition, O1w—H···S2 hydrogen bond is also found in the solid state.

### Experimental

All chemicals were of reagent grade quality obtained from commercial sources and used without further purification. Hexamethylenetetramine (0.50 mmol, 0.07 g), KSCN (1 mmol, 0.10 g) and Co(NO_3_)_2_.6H_2_O (0.50 mmol, 0.15 g) were mixed in methanol (25 ml).The resulting purple solution was left for few weeks at room temperature to afford purple crystals (yield 65%). Anal. Calcd. for [Co(hmt)(SCN)_2_(CH_3_OH)_2_(H_2_O)]: C 30.23, H 5.58, N 21.15%. Found: C 30.21, H 5.59, N 21.16%. IR (KBr pellet, cm ^-1^): 3398 (*m*), 2951 (*m*), 2877 (*m*), 2079 (*vs*), 1666 (*m*), 1462 (*s*), 1379 (*s*), 1241 (*s*), 1010 (*s*), 814 (*m*), 687 (*s*), 516 (*m*), 480 (*m*).

### Refinement

H atoms bonded to O atoms of CH_3_OH and H_2_O molecules were found in a difference map and refined freely. Other H atoms (hmt ligand) were generated geometrically and refined using a riding model: C—H = 0.97 Å, *U*_iso_(H) = 1.2 *U*_eq_(carrier C).

### Crystal data

**Table:**

[Co(NCS)_2_(C_6_H_12_N_4_)(CH_4_O)_2_(H_2_O)]	*F*_000_ = 1656
*M**_r_* = 397.39	*D*_x_ = 1.523 Mg m^−^^3^
Orthorhombic, *P**b**c**a*	Mo *K*α radiation λ = 0.71073 Å
Hall symbol: -P 2ac 2ab	Cell parameters from 7990 reflections
*a* = 14.1128 (8) Å	θ = 2.3–28.3º
*b* = 15.3684 (9) Å	µ = 1.25 mm^−^^1^
*c* = 15.9839 (9) Å	*T* = 296 (2) K
*V* = 3466.8 (3) Å^3^	Block, purple
*Z* = 8	0.30 × 0.30 × 0.25 mm

### Data collection

**Table:**

Bruker APEXII diffractometer	4287 independent reflections
Radiation source: sealed tube	3528 reflections with *I* > 2σ(*I*)
Monochromator: graphite	*R*_int_ = 0.022
*T* = 296(2) K	θ_max_ = 28.3º
φ and ω scans	θ_min_ = 2.3º
Absorption correction: multi-scan(SADABS; Bruker, 2000)	*h* = −14→18
*T*_min_ = 0.691, *T*_max_ = 0.730	*k* = −19→20
20785 measured reflections	*l* = −18→21

### Refinement

**Table:**

Refinement on *F*^2^	Secondary atom site location: difference Fourier map
Least-squares matrix: full	Hydrogen site location: inferred from neighbouring sites
*R*[*F*^2^ > 2σ(*F*^2^)] = 0.029	H atoms treated by a mixture of independent and constrained refinement
*wR*(*F*^2^) = 0.075	*w* = 1/[σ^2^(*F*_o_^2^) + (0.0359*P*)^2^ + 1.3724*P*] where *P* = (*F*_o_^2^ + 2*F*_c_^2^)/3
*S* = 1.05	(Δ/σ)_max_ = 0.001
4287 reflections	Δρ_max_ = 0.64 e Å^−^^3^
217 parameters	Δρ_min_ = −0.61 e Å^−^^3^
Primary atom site location: structure-invariant direct methods	Extinction correction: none

### Fractional atomic coordinates and isotropic or equivalent isotropic displacement parameters (Å^2^)

**Table:**

	*x*	*y*	*z*	*U*_iso_*/*U*_eq_	
Co1	0.553065 (15)	1.038335 (14)	0.283547 (14)	0.02795 (7)	
N1	0.65439 (11)	0.97495 (10)	0.35331 (10)	0.0373 (3)	
C1	0.71280 (12)	0.94096 (11)	0.39145 (11)	0.0309 (3)	
S1	0.79561 (4)	0.89311 (4)	0.44512 (4)	0.05772 (17)	
N2	0.45742 (11)	1.10745 (11)	0.21469 (10)	0.0412 (4)	
C2	0.40515 (12)	1.14790 (11)	0.17538 (10)	0.0318 (3)	
S2	0.33147 (4)	1.20788 (3)	0.12175 (3)	0.04690 (13)	
N3	0.43978 (9)	0.94296 (9)	0.32720 (8)	0.0262 (3)	
N4	0.27067 (9)	0.91344 (9)	0.34713 (9)	0.0310 (3)	
N5	0.38399 (10)	0.86701 (9)	0.45350 (9)	0.0329 (3)	
N6	0.37919 (10)	0.79376 (9)	0.31828 (9)	0.0320 (3)	
C3	0.34122 (11)	0.97472 (10)	0.31390 (11)	0.0295 (3)	
H3A	0.3336	1.0306	0.3412	0.035*	
H3B	0.3304	0.9829	0.2545	0.035*	
C4	0.45227 (12)	0.92901 (11)	0.41871 (10)	0.0311 (3)	
H4A	0.5159	0.9078	0.4291	0.037*	
H4B	0.4455	0.9843	0.4473	0.037*	
C5	0.44845 (11)	0.85686 (11)	0.28545 (10)	0.0301 (3)	
H5A	0.4387	0.8639	0.2258	0.036*	
H5B	0.5120	0.8344	0.2939	0.036*	
C6	0.28784 (12)	0.90063 (12)	0.43761 (10)	0.0359 (4)	
H6A	0.2798	0.9556	0.4665	0.043*	
H6B	0.2416	0.8600	0.4597	0.043*	
C7	0.28332 (12)	0.82913 (11)	0.30472 (11)	0.0341 (4)	
H7A	0.2726	0.8364	0.2452	0.041*	
H7B	0.2368	0.7881	0.3257	0.041*	
C8	0.39459 (13)	0.78357 (11)	0.40908 (11)	0.0355 (4)	
H8A	0.3493	0.7420	0.4312	0.043*	
H8B	0.4577	0.7607	0.4188	0.043*	
O1	0.52358 (11)	1.12213 (8)	0.39015 (8)	0.0416 (3)	
H1	0.5632 (16)	1.1230 (15)	0.4256 (16)	0.054 (7)*	
C9	0.4697 (2)	1.19973 (17)	0.39250 (16)	0.0775 (9)	
H9A	0.4301	1.1996	0.4413	0.116*	
H9D	0.4308	1.2033	0.3433	0.116*	
H9B	0.5116	1.2489	0.3945	0.116*	
O2	0.58559 (9)	0.95999 (9)	0.17962 (8)	0.0368 (3)	
H2	0.6396 (16)	0.9493 (13)	0.1762 (13)	0.039 (6)*	
C10	0.54971 (15)	0.97087 (17)	0.09672 (13)	0.0556 (6)	
H10D	0.5700	0.9230	0.0625	0.083*	
H10A	0.5733	1.0243	0.0737	0.083*	
H10B	0.4817	0.9726	0.0983	0.083*	
O1W	0.66088 (11)	1.12763 (9)	0.24788 (10)	0.0439 (3)	
H1WA	0.6425 (17)	1.1713 (16)	0.2319 (16)	0.054 (7)*	
H1WB	0.7014 (18)	1.1426 (16)	0.2873 (16)	0.063 (8)*	

### Atomic displacement parameters (Å^2^)

**Table:**

	*U*^11^	*U*^22^	*U*^33^	*U*^12^	*U*^13^	*U*^23^
Co1	0.02528 (12)	0.02913 (12)	0.02945 (12)	0.00166 (8)	−0.00442 (8)	0.00671 (8)
N1	0.0328 (8)	0.0382 (8)	0.0408 (8)	0.0019 (6)	−0.0058 (6)	0.0072 (6)
C1	0.0299 (8)	0.0296 (8)	0.0333 (8)	−0.0020 (6)	−0.0040 (7)	−0.0008 (6)
S1	0.0564 (3)	0.0461 (3)	0.0707 (4)	0.0114 (2)	−0.0366 (3)	−0.0015 (3)
N2	0.0362 (8)	0.0436 (9)	0.0437 (9)	0.0042 (7)	−0.0059 (7)	0.0111 (7)
C2	0.0306 (8)	0.0336 (8)	0.0314 (8)	−0.0002 (7)	−0.0020 (7)	0.0022 (7)
S2	0.0437 (3)	0.0503 (3)	0.0468 (3)	0.0126 (2)	−0.0123 (2)	0.0089 (2)
N3	0.0253 (6)	0.0283 (7)	0.0251 (6)	−0.0001 (5)	−0.0025 (5)	0.0014 (5)
N4	0.0265 (7)	0.0330 (7)	0.0336 (7)	0.0002 (5)	0.0006 (6)	−0.0010 (6)
N5	0.0338 (7)	0.0373 (8)	0.0277 (7)	−0.0022 (6)	0.0008 (6)	0.0043 (6)
N6	0.0356 (7)	0.0268 (7)	0.0336 (7)	−0.0008 (6)	0.0021 (6)	−0.0015 (6)
C3	0.0284 (8)	0.0286 (8)	0.0314 (8)	0.0024 (6)	−0.0020 (6)	0.0011 (6)
C4	0.0327 (9)	0.0363 (9)	0.0243 (7)	−0.0034 (7)	−0.0044 (6)	0.0020 (6)
C5	0.0307 (8)	0.0299 (8)	0.0297 (8)	0.0017 (6)	0.0031 (6)	−0.0018 (6)
C6	0.0335 (9)	0.0435 (10)	0.0308 (8)	0.0009 (7)	0.0065 (7)	−0.0010 (7)
C7	0.0314 (9)	0.0346 (9)	0.0364 (9)	−0.0058 (7)	−0.0025 (7)	−0.0032 (7)
C8	0.0379 (9)	0.0310 (8)	0.0377 (9)	−0.0003 (7)	0.0013 (7)	0.0075 (7)
O1	0.0519 (8)	0.0371 (7)	0.0358 (7)	0.0080 (6)	−0.0109 (6)	−0.0021 (5)
C9	0.116 (2)	0.0597 (15)	0.0565 (15)	0.0428 (15)	−0.0207 (15)	−0.0112 (12)
O2	0.0253 (6)	0.0531 (8)	0.0319 (6)	0.0018 (5)	−0.0003 (5)	0.0017 (5)
C10	0.0418 (12)	0.0907 (18)	0.0343 (10)	0.0045 (11)	−0.0045 (8)	0.0006 (10)
O1W	0.0411 (8)	0.0344 (7)	0.0562 (9)	−0.0044 (6)	−0.0076 (7)	0.0132 (7)

### Geometric parameters (Å, °)

**Table:**

Co1—N2	2.0400 (15)	C3—H3B	0.9700
Co1—N1	2.0585 (15)	C4—H4A	0.9700
Co1—O2	2.1024 (13)	C4—H4B	0.9700
Co1—O1W	2.1268 (14)	C5—H5A	0.9700
Co1—O1	2.1760 (13)	C5—H5B	0.9700
Co1—N3	2.2785 (13)	C6—H6A	0.9700
N1—C1	1.151 (2)	C6—H6B	0.9700
C1—S1	1.6256 (17)	C7—H7A	0.9700
N2—C2	1.151 (2)	C7—H7B	0.9700
C2—S2	1.6327 (17)	C8—H8A	0.9700
N3—C5	1.487 (2)	C8—H8B	0.9700
N3—C4	1.489 (2)	O1—C9	1.415 (2)
N3—C3	1.489 (2)	O1—H1	0.80 (2)
N4—C3	1.470 (2)	C9—H9A	0.9600
N4—C7	1.473 (2)	C9—H9D	0.9600
N4—C6	1.480 (2)	C9—H9B	0.9600
N5—C4	1.465 (2)	O2—C10	1.428 (2)
N5—C8	1.473 (2)	O2—H2	0.78 (2)
N5—C6	1.474 (2)	C10—H10D	0.9600
N6—C5	1.473 (2)	C10—H10A	0.9600
N6—C7	1.474 (2)	C10—H10B	0.9600
N6—C8	1.476 (2)	O1W—H1WA	0.76 (3)
C3—H3A	0.9700	O1W—H1WB	0.88 (3)
			
N2—Co1—N1	176.69 (6)	N6—C5—N3	111.79 (12)
N2—Co1—O2	90.94 (6)	N6—C5—H5A	109.3
N1—Co1—O2	90.31 (6)	N3—C5—H5A	109.3
N2—Co1—O1W	89.59 (6)	N6—C5—H5B	109.3
N1—Co1—O1W	87.34 (6)	N3—C5—H5B	109.3
O2—Co1—O1W	90.09 (6)	H5A—C5—H5B	107.9
N2—Co1—O1	89.30 (6)	N5—C6—N4	111.46 (13)
N1—Co1—O1	89.35 (6)	N5—C6—H6A	109.3
O2—Co1—O1	178.06 (6)	N4—C6—H6A	109.3
O1W—Co1—O1	87.99 (6)	N5—C6—H6B	109.3
N2—Co1—N3	92.06 (6)	N4—C6—H6B	109.3
N1—Co1—N3	90.98 (5)	H6A—C6—H6B	108.0
O2—Co1—N3	91.53 (5)	N4—C7—N6	111.58 (13)
O1W—Co1—N3	177.67 (6)	N4—C7—H7A	109.3
O1—Co1—N3	90.39 (5)	N6—C7—H7A	109.3
C1—N1—Co1	178.20 (15)	N4—C7—H7B	109.3
N1—C1—S1	179.8 (2)	N6—C7—H7B	109.3
C2—N2—Co1	178.33 (16)	H7A—C7—H7B	108.0
N2—C2—S2	178.14 (17)	N5—C8—N6	111.51 (13)
C5—N3—C4	107.64 (13)	N5—C8—H8A	109.3
C5—N3—C3	107.72 (12)	N6—C8—H8A	109.3
C4—N3—C3	107.34 (12)	N5—C8—H8B	109.3
C5—N3—Co1	112.16 (9)	N6—C8—H8B	109.3
C4—N3—Co1	108.08 (9)	H8A—C8—H8B	108.0
C3—N3—Co1	113.63 (9)	C9—O1—Co1	128.49 (13)
C3—N4—C7	108.37 (13)	C9—O1—H1	110.2 (17)
C3—N4—C6	109.12 (13)	Co1—O1—H1	115.6 (17)
C7—N4—C6	108.23 (13)	O1—C9—H9A	109.5
C4—N5—C8	108.45 (13)	O1—C9—H9D	109.5
C4—N5—C6	108.18 (13)	H9A—C9—H9D	109.5
C8—N5—C6	108.39 (14)	O1—C9—H9B	109.5
C5—N6—C7	108.29 (13)	H9A—C9—H9B	109.5
C5—N6—C8	108.81 (13)	H9D—C9—H9B	109.5
C7—N6—C8	108.60 (13)	C10—O2—Co1	126.06 (13)
N4—C3—N3	111.79 (12)	C10—O2—H2	107.8 (16)
N4—C3—H3A	109.3	Co1—O2—H2	112.9 (16)
N3—C3—H3A	109.3	O2—C10—H10D	109.5
N4—C3—H3B	109.3	O2—C10—H10A	109.5
N3—C3—H3B	109.3	H10D—C10—H10A	109.5
H3A—C3—H3B	107.9	O2—C10—H10B	109.5
N5—C4—N3	112.87 (13)	H10D—C10—H10B	109.5
N5—C4—H4A	109.0	H10A—C10—H10B	109.5
N3—C4—H4A	109.0	Co1—O1W—H1WA	114.4 (19)
N5—C4—H4B	109.0	Co1—O1W—H1WB	116.1 (16)
N3—C4—H4B	109.0	H1WA—O1W—H1WB	103 (2)
H4A—C4—H4B	107.8		

### Hydrogen-bond geometry (Å, °)

**Table:**

*D*—H···*A*	*D*—H	H···*A*	*D*···*A*	*D*—H···*A*
C4—H4A···N1	0.97	2.52	3.119 (2)	120
C4—H4B···O1	0.97	2.56	3.167 (2)	121
C9—H9D···N2	0.96	2.56	3.181 (3)	123
O1—H1···N5^i^	0.80 (2)	2.08 (3)	2.824 (2)	156 (2)
O1W—H1WA···N6^ii^	0.76 (2)	2.07 (2)	2.821 (2)	168 (3)
O2—H2···N4^iii^	0.78 (2)	1.97 (2)	2.7417 (18)	172 (2)
O1W—H1WB···S2^iii^	0.88 (3)	2.55 (3)	3.4146 (16)	168 (2)

Symmetry codes: (i) −*x*+1, −*y*+2, −*z*+1; (ii) −*x*+1, *y*+1/2, −*z*+1/2; (iii) *x*+1/2, *y*, −*z*+1/2.
